# Development and Validation of a Comprehensive Prognostic and Depression Risk Index for Gastric Adenocarcinoma

**DOI:** 10.3390/ijms251910776

**Published:** 2024-10-07

**Authors:** Sheng Tian, Yixin Liu, Pan Liu, Sachiyo Nomura, Yongchang Wei, Tianhe Huang

**Affiliations:** 1 Department of Radiation and Medical Oncology, Zhongnan Hospital of Wuhan University, Wuhan University, Wuhan 430071, China; 2016302180097@whu.edu.cn (S.T.); yixinliu@whu.edu.cn (Y.L.); liupanzn@whu.edu.cn (P.L.); 2 Hubei Key Laboratory of Tumor Biological Behaviors, Zhongnan Hospital of Wuhan University, Wuhan University, Wuhan 430071, China; 3 Hubei Cancer Clinical Study Center, Zhongnan Hospital of Wuhan University, Wuhan University, Wuhan 430071, China; 4 Department of Gastrointestinal Surgery, Graduate School of Medicine, The University of Tokyo, 7-3-1 Hongo, Bunkyo-ku, Tokyo 113-8655, Japan; sachiyo.nomura1012@gmail.com

**Keywords:** gastric adenocarcinoma, depression, gene signature, prognosis, neurogenesis

## Abstract

Depressive disorder contributes to the initiation and prognosis of patients with cancer, but the interaction between cancer and depressive disorder remains unclear. We generated a gastric adenocarcinoma patient-derived xenograft mice model, treated with chronic unpredictable mild stimulation. Based on the RNA-sequence from the mouse model, patient data from TCGA, and MDD-related (major depressive disorder) genes from the GEO database, 56 hub genes were identified by the intersection of differential expression genes from the three datasets. Molecular subtypes and a prognostic signature were generated based on the 56 genes. A depressive mouse model was constructed to test the key changes in the signatures. The signature was constructed based on the NDUFA4L2, ANKRD45, and AQP3 genes. Patients with high risk-score had a worse overall survival than the patients with low scores, consistent with the results from the two GEO cohorts. The comprehensive results showed that a higher risk-score was correlated with higher levels of tumor immune exclusion, higher infiltration of M0 macrophages, M2 macrophages, and neutrophils, higher angiogenetic activities, and more enriched epithelial–mesenchymal transition signaling pathways. A higher risk score was correlated to a higher MDD score, elevated MDD-related cytokines, and the dysfunction of neurogenesis-related genes, and parts of these changes showed similar trends in the animal model. With the Genomics of Drug Sensitivity in Cancer database, we found that the gastric adenocarcinoma patients with high risk-score may be sensitive to Pazopanib, XMD8.85, Midostaurin, HG.6.64.1, Elesclomol, Linifanib, AP.24534, Roscovitine, Cytarabine, and Axitinib. The gene signature consisting of the NDUFA4L2, ANKRD45, and AQP3 genes is a promising biomarker to distinguish the prognosis, the molecular and immune characteristics, the depressive risk, and the therapy candidates for gastric adenocarcinoma patients.

## 1. Introduction

In the past few decades, the cross-talk between depressive disorder and cancer has been widely evaluated, though it remains controversial. Wang et al. confirmed that depressive disorder was associated with a significantly increased risk of cancer incidence and cancer-specific mortality [1]. Several studies with animal models provided solid evidence that depressive disorder facilitated tumor growth and metastasis [2,3,4]. Notably, depressive disorder contributes to cancer incidence and cancer progression. Meanwhile, studies confirmed that cancer diagnosis and treatment induced physical and cognitive changes in cancer patients [5]. We could conclude that there is an exact interaction between cancer and depression. Immunomodulation and inflammation were recognized as the driving forces for both depression and cancer [6,7]. However, the immune components and inframammary factors vary in different cancer types. The complex changes during the interaction make it hard to take effective interventions for cancer patients with depression.

In our previous work, we discovered that depression accelerated the tumorigenesis of gastric adenocarcinoma, and gastric adenocarcinoma patients with depression experienced worse overall survival (OS). The potential mechanism might lie in the inflammation induced by the ABL1 [3]. In our following work, we tried to target gastric cancer patients with depression by targeting ABL1, but unfortunately failed. Therefore, we considered establishing the hallmarks of gastric cancer patients with depression. In this study, we tried to develop a gene signature for gastric adenocarcinoma with depression, which could predict the prognosis and depression risk, and offer suggestions for personalized pharmacological and psychosocial approaches. We focused on the molecular and immune characteristics, enriched signaling pathways, and neurogenesis makers in patients identified by the gene signature.

## 2. Results

### 2.1. Major Depressive Disorder and Gastric Adenocarcinoma-Related Hub Genes

**Figure 1:**
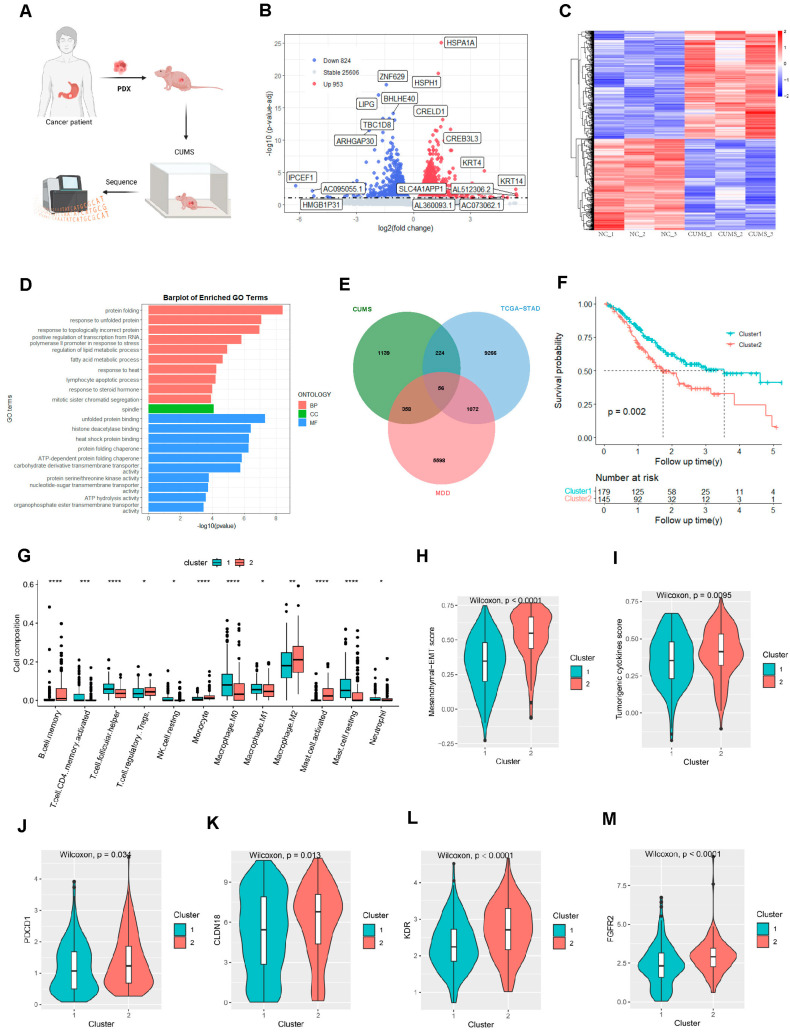

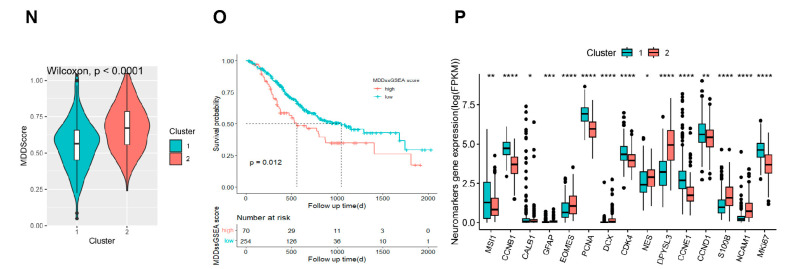
Major depressive disorder- and gastric adenocarcinoma-related hub genes (**A**) Workflow for the construction of the gastric adenocarcinoma PDX mice model treated with CUMS and gene profiling. (**B**) A visualization of the RNA-Seq results with Volcano Plot for PDX vs PDX with CUMS. (**C**) A heatmap of the differential genes’ expression between PDX vs PDX with CUMS. (**D**) A barplot of the enriched GO terms (including Biological Process, Cellular Component, and Molecular Function) for the differential genes between PDX vs PDX with CUMS. (**E**) The intersection of differential genes from MDD, PDX with CUMS, and the TCGA STAD cohort. (**F**) A survival analysis determined by K–M for the two clusters divided by the 56 genes in the STAD dataset. (**G**) The composition of infiltrating immune cells in the two clusters using CIBERSORT. (**H**,**I**) A violin plot of the ssGSEA score of EMT and tumorigenic cytokines in the two clusters. (**J**–**M**) A violin plot of the expression levels of PDCD1, CLDN18, KDR, and FGFR2 in the two clusters. (**N**) A violin plot of the MDD score in the two clusters. (**O**) A survival analysis determined by K–M for the group divided by MDD score in the STAD TCGA database; the cutoff MDD score was obtained by X-tile. (**P**) The relative expression levels of the neurogenesis markers in the two clusters. The results are presented as mean ± standard deviation (SD). * *p* < 0.05, ** *p* < 0.01, *** *p* < 0.001, and **** *p* < 0.0001, two-sided, Wilcoxon test.

To establish the hallmarks of gastric adenocarcinoma patients with depression, we employed the gastric adenocarcinoma patient-derived xenograft mice model treated with CUMS stimulation (Figure 1A). In differential expression analysis (3 PDX vs. 3 PDX with CUMS), a total of 1777 DEGs were obtained, of which 953 genes were upregulated and 824 genes were downregulated in the CUMS-treated samples compared with the control (Figure 1B and Table S1). These two groups showed different gene expression patterns (Figure 1C) and these genes were mainly enriched in the cell division process and metabolic remodeling signaling pathways (Figure 1D). The gene profiling of the patients from TCGA STAD dataset was summarized (Figure S1a,b). Cytoscape was used to visualize the DEGs’ PPI network obtained from the STRING database (Figure S1c). Take the intersection of these genes with previously studied depression-related genes [8] and tumor-related genes obtained from TCGA STAD as the hub genes (Figure 1E). Cluster analysis was utilized based on the 56 hub genes. The optimal clustering number is 2, and the two subgroups of STAD patients show good internal consistency and stability (Figure S1d–f). Cluster 1 has a better prognosis than cluster 2 (Figure 1F). Next, we investigated the composition of immune cells in different subgroups using the CIBERSORT algorithm, and we used the Wilcoxon test to compare the distribution of immune cells. The result showed that B memory cells, T regulatory cells, monocyte, M2 macrophages, and activated mast cells were more abundant in cluster 2, while activated memory CD4 T cells, T helper cells, resting natural killer cells, M0 macrophages, M1 macrophages, resting mast cells, and neutrophils were less abundant in cluster 2 (Figure 1G). The heatmap showed the different gene expression patterns between the two clusters (Figure S1g). With TIDE and ESTIMATE scores, we confirmed that patients in cluster 2 had an immune suppressive phenotype (Figure S1h–k). Interestingly, cluster 2 showed suppressed levels of ERBB2, VEGFA, and angiogenic activity score (Figure S1l–n). Regarding the molecular characters for the tumor cells, we confirmed that cluster 2 exhibited an enriched EMT score (Figure 1H) and tumor cytokines score (Figure 1I). The immune checkpoint gene PDCD1, with other target genes including CLAUDIN 18, KDR, and FGFR2, were dramatically elevated in the patients in cluster 2 (Figure 1J–M). Surprisingly, we constructed a major depressive disorder score (MDD score) and confirmed that patients in cluster 2 experienced high MDD scores (Figure 1N). The higher MDD scores were confirmed to be negatively correlated to the OS of gastric adenocarcinoma patients from the TCGA dataset (Figure 1O). Since neurodegeneration was thought to involve in the depressive disorder, the expression of several neurogenesis markers was detected in the subgroups. We confirmed that MSI1, CCNB1, CABL1, PCNA, CDK4, CCNE1, CCND1, and MKI-67 were significantly suppressed, and GFAP, EOMES, DCX, NES, DPYSL3, S100B, and NCAM1 were notably increased in cluster 2 (Figure 1P). The dysfunction of the neurogenesis-related genes may explain why patients in clusters are susceptible to depressive disorder. The data here suggested that the 56-gene-based gene signature is associated with the OS, immune phenotype, molecular characteristics, and the depressive risk of gastric adenocarcinoma patients.

### 2.2. The Gene Signature Based on the NDUFA4L2, ANKRD45, and AQP3 Predicts the Prognosis of Gastric Adenocarcinoma

**Figure 2:**
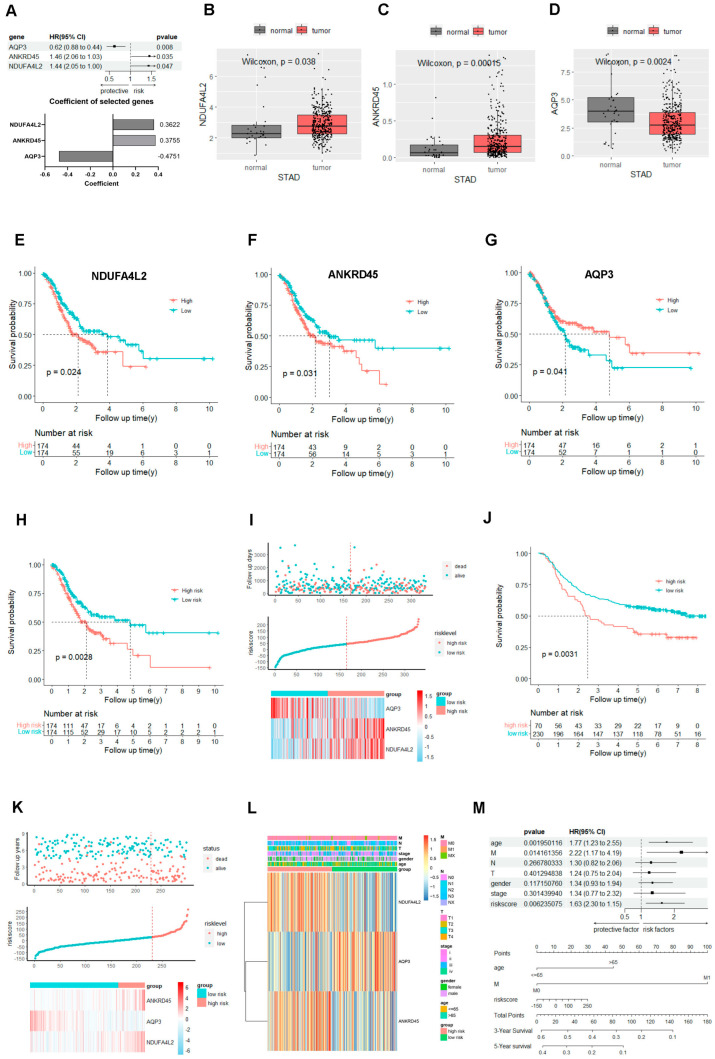
The gene signature based on the NDUFA4L2, ANKRD45, and AQP3 predicts the prognosis of gastric adenocarcinoma (**A**) Forest plot and risk-score coefficient for three independent prognostic genes (NDUFA4L2, ANKRD45, and AQP3) determined by multivariate Cox regression analysis. (**B**–**D**) A comparison of NDUFA4L2, ANKRD45, and AQP3 in normal tissue and gastric tumor tissue in the TCGA cohort. (**E**–**G**) A survival analysis determined by K–M for the NDUFA4L2, ANKRD45, and AQP3 genes in TCGA cohort, grouped according to the median of the expression value. (**H**,**I**) A survival analysis, heatmap, and survival status accompanied by the risk score in TCGA cohort, grouped according to the median of the risk score. (**J**,**K**) A survival analysis, heatmap, and survival status accompanied by the risk score in the GSE84437 cohort, grouped according to the best risk-score obtained by X-tile. (**L**) A heatmap of the association between the expression levels of the three genes and clinicopathological features. (**M**) The signature was an independent risk factor for STAD patients in TCGA cohort according to multivariate Cox analysis, and a nomogram based on risk score, age, and M stage. The results are presented as mean ± standard deviation (SD). * *p* < 0.05, ** *p* < 0.01, *** *p* < 0.001, and **** *p* < 0.0001, , two-sided, Wilcoxon test.

Three independent prognostic genes (NDUFA4L2, ANKRD45, and AQP3) were determined by multivariate Cox regression analysis for OS among the 56 hub genes. Then, we constructed a gene signature calculated by the coefficients of 0.3622, 0.3755, and −0.4751 for NDUFA4L2, ANKRD45, and AQP3, respectively (Figure 2A). For the individual gene, NDUFA4L2 and ANKRD45 were upregulated in the gastric adenocarcinoma (Figure 2B,C), and were negatively correlated with the OS (Figure 2E,F). AQP3 was downregulated in the gastric adenocarcinoma (Figure 2D) and was positively correlated with the OS (Figure 2G). The signature based on the NDUFA4L2, ANKRD45, and AQP3 genes was evaluated with the area under curve (AUC) and nomogram-predicted probability (Figure S2a–c), and the signature showed an ideal value to predict the OS of gastric adenocarcinoma. Patients with high risk-score experienced decreased OS (Figure 2H,I). The signature was validated in two GEO gastric adenocarcinoma datasets and predicted the OS of the patients very well in the GSE84437 (Figure 2J,K) and GSE62254 (Figure S2g,h). The general characteristics of the patients in TCGA and two GEO cohorts were shown in Table S3. The relationship between NDUFA4L2, ANKRD45, and AQP3 with the clinicopathologic characteristics of gastric adenocarcinoma was presented with the heatmap (Figure 2L). Combined with the clinicopathologic characteristics of 333 patients with gastric adenocarcinoma in TCGA, we identified that age, M stage, and risk score calculated by the signature are independent prognostic factors by multivariate Cox regression analysis. A nomogram was constructed including age, M stage, and the signature, which is based on the multivariate Cox regression analysis results (Figure 2M). Calibration plots showed a high consistency between the actual survival times and the predicted survival times (Figure 2D–F).

### 2.3. The Signature Based on the NDUFA4L2, ANKRD45, and AQP3 Defined the Molecular Characteristics and the Tumor Microenvironment of Gastric Adenocarcinoma

**Figure 3:**
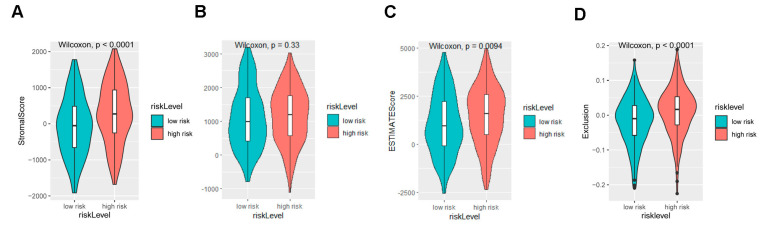

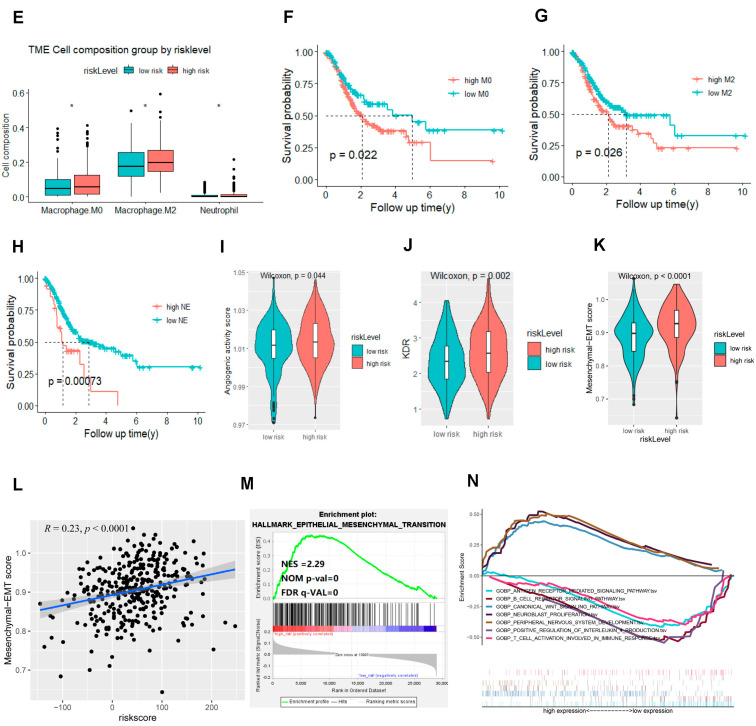
The signature based on the NDUFA4L2, ANKRD45, and AQP3 defined the molecular characteristics and the tumor microenvironment of gastric adenocarcinoma (**A**–**C**) Immune and stromal scores using ESTIMATE between the high- and low-risk groups in TCGA STAD cohort. (**D**) TIDE score between the high- and low-risk groups. (**E**) The composition of infiltrating immune cells using CIBERSORT. (**F**–**H**) A survival analysis determined by K–M for the infiltration of M0 macrophages, M2 macrophage, and the neutrophils in the TCGA STAD database, grouped according to the CIBERSORT’s result. (**I**,**K**) A violin plot of the ssGSEA score of angiogenic and EMT between the high- and low-risk groups. (**J**) A violin plot of the KDR level. (**L**) A correlation analysis between the EMT score and the risk-score in the STAD dataset. (**M**,**N**) The top enriched terms in high-risk or low-risk groups using GSEA. The results are presented as mean ± standard deviation (SD). * *p* < 0.05, two-sided, Wilcoxon test.

Given that the signature had the robust capacity to predict the OS, we next tried to uncover the potential mechanism leading to the outcome for the subgroups divided by the signature. We analyzed the molecular characteristics and the tumor microenvironment of the subgroups. The subgroup with a high risk-score identified by the signature based on the three genes had a higher stromal score and exclusion score, which indicates an immune suppressive phenotype (Figure 3A–D). For the composition of immune cells, macrophage M0, macrophage M2, and neutrophils are the dominant raising cell populations in the subgroups with high scores (Figure 3E). Interestingly, high infiltration levels of macrophage M0, macrophage M2, and neutrophils were associated with worse OS, respectively (Figure 3F–H). For the characters of the tumor cells, we observed notably enriched angiogenic activities and an overexpression of KDR (Figure 3I,J). Another significantly enriched signaling pathway was the EMT signaling pathway (Figure 3K–M), which defined the subgroup with a high score as more aggressive behavior. With GSEA, we found most cancer-related signaling pathways were enriched in the subgroup (Figure 3N), of which canonical WNT signaling pathways, neuroblast proliferation, and peripheral nervous system development are activated in the subgroup with a high risk-score. There are several suppressed pathways including antigen receptor and B cell receptor signaling pathways, the pathways of the positive regulation of IL-4 production and T cell activation involved in immune response. The enriched cytokines’ regulation, immune cell regulation, and WNT pathways may function to drive the immuno-suppressive tumor microenvironment (TME) and aggressive behaviors of gastric adenocarcinoma.

### 2.4. The Signature Based on the NDUFA4L2, ANKRD45, and AQP3 Estimated the Depressive Risk of Gastric Adenocarcinoma

**Figure 4:**
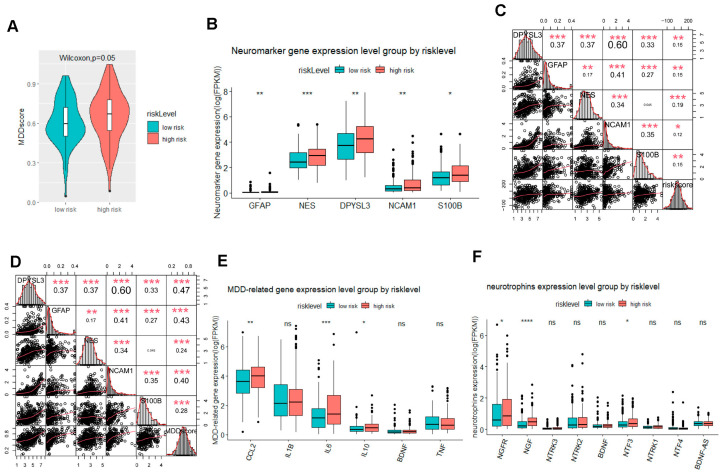

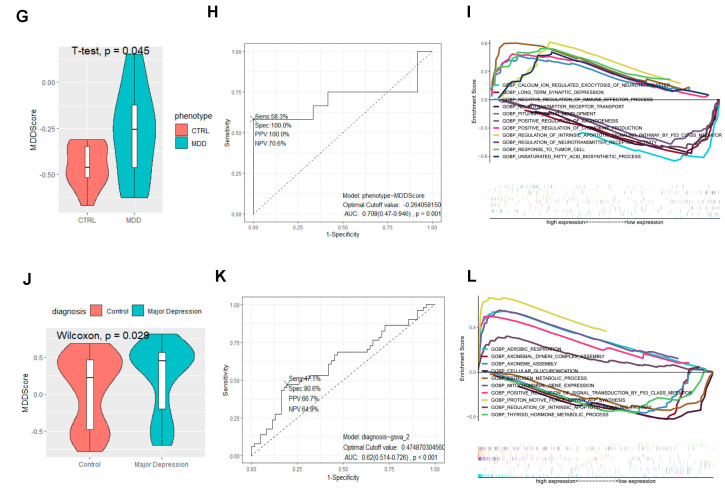
The signature based on the NDUFA4L2, ANKRD45, and AQP3 estimated the depressive risk of gastric adenocarcinoma (**A**) A violin plot of the MDD score in the subgroups divided by risk-score in the STAD dataset. (**B**) The levels of neurogenesis markers in the subgroups in the STAD dataset. (**C**) A correlation analysis between the neurogenesis markers and the risk-score in the STAD dataset. (**D**) A correlation analysis between the neurogenesis markers and the MDD score in the STAD dataset. (**E**) The levels of MDD-related cytokines in the subgroups divided by the risk score in the STAD dataset. (**F**) The levels of neuron growth factors and receptors in the subgroups divided by the risk score in the STAD dataset. (**G**) A violin plot of MDD score in MDD patients in the GEO MDD dataset (GSE102556). (**H**) The ROC of the MDD score in the GEO MDD dataset (GSE102556). (**I**) The top enriched GO terms in the high-risk or low-risk group using GSEA. (**J**) A violin plot of the MDD score in MDD patients in the GEO MDD dataset (GSE80655). (**K**) The ROC of the MDD score in the GEO MDD dataset (GSE80655). (**L**) The top enriched GO terms in high-risk or low-risk group using GSEA. The results are presented as mean ± standard deviation (SD). * *p* < 0.05, ** *p* < 0.01, *** *p* < 0.001, and **** *p* < 0.0001, ns, not significant, two-sided, Wilcoxon test.

Since the genes were derived from the patients with MDD, we next tried to assess the predictive value for the depressive risk of gastric adenocarcinoma patients. With the gene-profiling data from the patients with MDD, we generated the MDD scores. Interestingly, we confirmed that the patients with high risk-score had significantly higher MDD scores (Figure 4A). Importantly, several neurogenesis-related genes, including GFAP, NES, DPYSL3, S100B, and NCAM1, were dramatically activated in the subgroup with high risk-score (Figure 4B). The correlation analysis revealed that neurogenesis-related genes were positively associated with the risk score and MDD score (Figure 4C,D). Several depression-related cytokines including CCL2, IL-6, and IL-10 were significantly increased (Figure 4E). Surprisingly, tumor-derived neurotrophins and neuron growth factor receptors notably increased in the subgroup with high risk-score (Figure 4F). To better evaluate the predictive value of the MDD score, we applied the MDD score in two MDD-related GEO datasets and confirmed that the MDD score was significantly higher in the MDD patients, with a specificity of 100% in the GSE102556 (Figure 4G,H). The alteration of signal pathways in MDD included activation of the negative regulation of the immune effector process, the regulation of the intrinsic apoptotic signaling pathway by p53 class mediator, response to tumor cell, and the positive regulation of chemokine production pathways, as well as the inhibited positive regulation of axonogenesis, the calcium-ion-regulated exocytosis of a neurotransmitter, and the long-term synaptic depression pathways (Figure 4I). The targets detected in the dataset GSE80655 showed a similar trend with the GSE102556 even without ideal specificity and sensitivity (Figure 4J,K), suggesting that our gene signature showed promising specificity to predict the MDD. The analysis of the signaling pathways discovered the dysfunction of metabolic and axoneme-assembly-related pathways (Figure 4L). The data here suggested that gastric adenocarcinoma patients with a high risk-score are susceptible to depression. The dysregulated neurogenesis-related genes, cytokines, and immune factors may contribute to the depressive risk for gastric adenocarcinoma patients.

### 2.5. Signature Genes’ Expression and Immune Infiltration in Gastric Cancer Following CUMS Treatment

**Figure 5:**
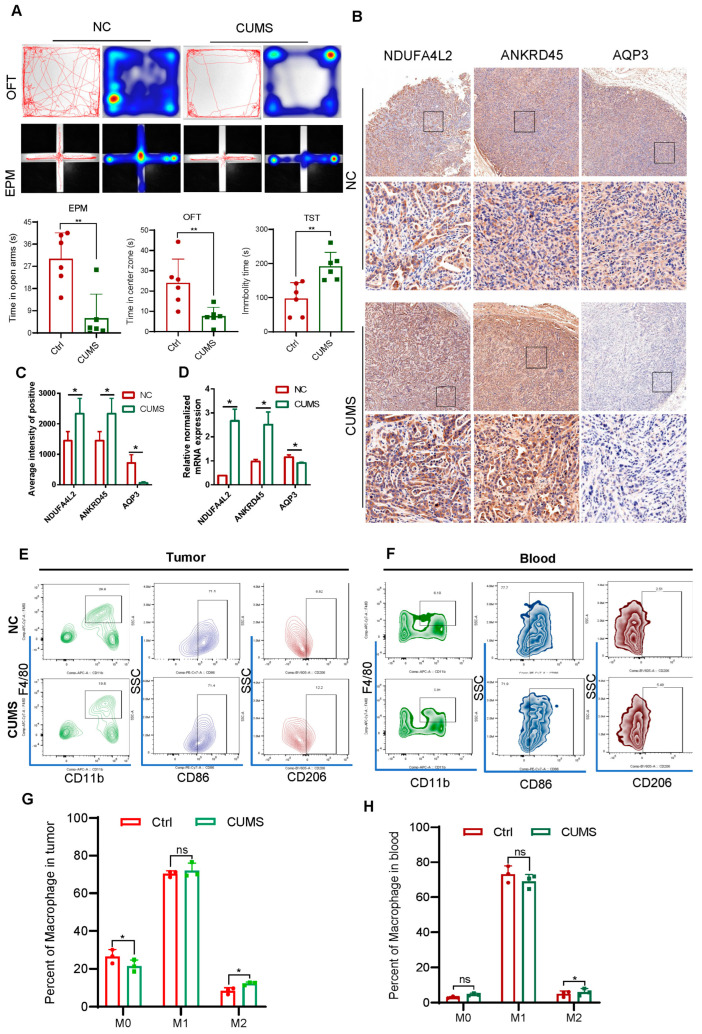
Signature genes’ expression and immune infiltration in gastric cancer following CUMS treatment (**A**) Representative signature genes’ IHC staining of tumor tissue from control and CUMS group (n = 3). (**B**,**C**) The quantification of IHC chromogen stain intensity for signature genes in the control and CUMS group tumor tissue (n = 3). (**D**) The mRNA levels of signature genes were assessed in tumor tissue from control and CUMS group using RT–PCR (n = 3). (**E**–**H**) The percentage of different subtypes of macrophages in tumor and blood determined by flow cytometry (n = 3). *p* values determined by two-tailed unpaired *t*-test. Data are represented as the mean ± SD. * *p* < 0.05, ** *p* < 0.01, ns, not significant.

To further investigate the relationship between the signature and depressive or immune infiltration, we established a subcutaneous gastric tumor mouse model treated with CUMS. After 4 weeks of CUMS treatment, the mice showed clear depressive tendencies, demonstrated by reduced time spent in the center zone and open arms, and increased immobility duration in the tail suspension test (Figure 5A). The IHC signals for NDUFA4L2 and ANKRD45 were stronger in tumor tissue treated with CUMS, while AQP3 expression was weaker compared to the control (Figure 5B,C), and the mRNA levels of signature genes are consistent with the IHC results (Figure 5D). This trend aligns with the model parameters, suggesting that the CUMS group risk score is higher than that of the control group. Additionally, an analysis of macrophage infiltration revealed that CUMS treatment correlated with an increased infiltration of M2-like macrophages in tumor tissue, and a similar trend was observed in the blood (Figure 5E–H). Collectively, these results suggested that the signature was associated with depression and immune infiltration.

### 2.6. Potential Treatment Strategy for the Signature Defined Gastric Adenocarcinoma Patients

**Figure 6:**
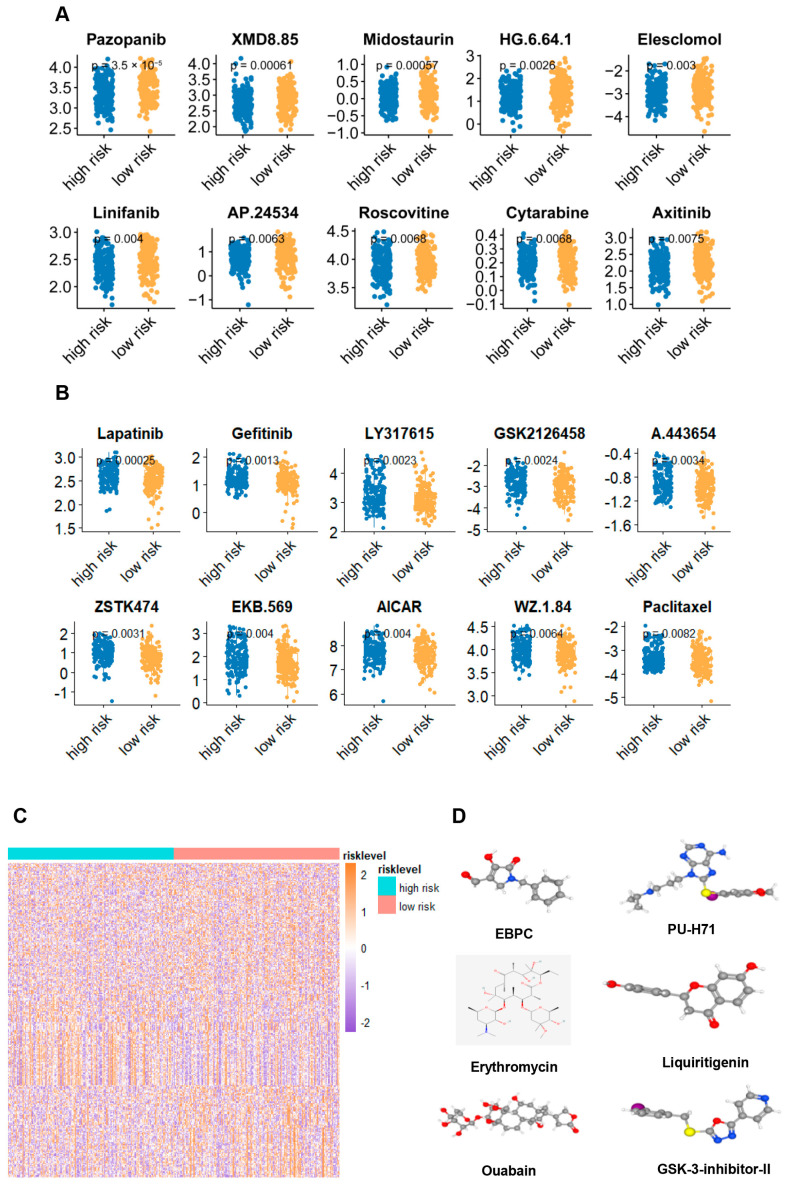
Potential treatment strategy for the signature defined gastric adenocarcinoma patients (**A**) Sensitive drugs screening for this subgroup with high risk score determined by the Genomics of Drug Sensitivity in Cancer database. (**B**) Sensitive drugs screening for this subgroup with low risk score determined by the Genomics of Drug Sensitivity in Cancer database. (**C**) Differentially expressed genes between the high- and low-risk groups in the TCGA cohort. (**D**) The structure of six potential target drugs screened from the cMap database.

Given that the signature clearly defined the group of gastric cancer patients with immuno-suppressive TME, aggressive behaviors, and depressive risk, we tried to uncover a potential treatment strategy for the group. Firstly, we reorganized the gene profiling of this group. Based on the genomics testing, we tried to find the potential sensitive drugs for this group with the Genomics of Drug Sensitivity in Cancer database. Surprisingly, we found the patients with a high score may be sensitive to drugs such as Pazopanib, XMD8.85, Midostaurin, HG.6.64.1, Elesclomol, Linifanib, AP.24534, Roscovitine, Cytarabine, and Axitinib (Figure 6A), and may be resistant to drugs like Lapatinib, Gefitinib, LY317615, GSK2126458, A.443654, ZSTK474, EKB.569, AICAR, W2.1.84, and Paclitaxel (Figure 6B). Moreover, with the gene expression files, we also utilized the Cmap dataset to find the potential inhibitor for enriched signaling pathways in the group and showed that EBPC, PU-H71, erythromycin, liquiritigenin, ouabain, and GSK-3-inhibitor-II maybe serve as the small molecular inhibitors for this subgroup (Figure 6C,D).

## 3. Materials and Methods

### 3.1. PDX Mouse Experiments

The specific pathogen-free (SPF) standards were followed in animal experiments and conformed to the National Institutes of Health Animal Use Guidelines. All the mice were purchased from Beijing Vital River Laboratory Animal Technology Co., Ltd. (Beijing, China). For gastric cancer patient-derived xenograft (PDX) tumor model, the surgical tissue fragments (from a 77-year-old female gastric adenocarcinoma patient without depression disorder in Zhongnan Hospital of Wuhan University) (approximately 10–15 mm^3^) were subcutaneously and directly implanted into female NOD-SCID (6-week-old) mice. The PDX tumors were propagated in the abdomen and axilla on both sides of the mice. After three successive transplantations, the xenografts were isolated and implanted into the right axilla of BALB-c/nu female mice (6–8-week-old).

Chronic unpredictable mild stress (CUMS) was applied to construct a model of depression in BALB/c nude mice. Specifically, a week after tumor transplantation, the mice were randomly divided into two groups (PDX, PDX with CUMS). The mice in group PDX with CUMS received several stressors, including being restrained in a confined space, tilting the cage 45 degrees, wet bedding, bedding deprivation, light/dark cycle inversion, food or water deprivation, and tail nipping. Each mouse was randomly treated with one of the stressors at different times of the day, and the same stressor was not repeated for seven days to reduce the predictability of each stressor. The mice in the PDX group were not exposed to any stressors and were allowed to freely eat and drink. Three weeks later, the depression status of mice was assessed by several behavioral tests as described before [3].

### 3.2. Subcutaneous Tumor Mouse Model

The C57BL/6 female mice (6–8 weeks of age) were purchased from the Hubei BIONT Biological Technology (Wuhan, China). A model of depression was established using the (CUMS) method, as described above. For the gastric cancer model, YTN3 cell lines were suspended in 50% Matrigel and subcutaneously implanted (8 × 10^6^ cells per tumor) into C57BL/6 mouse, and stress exposure was started the day after cancer cell injection and lasted for 4 weeks. The behavioral data were collected and analyzed using a computerized video tracking system (Ethovision XT 17.5, Noldus, Leesburg, VA, USA). At the endpoint of the experiment, single-cell suspensions were collected and isolated from blood and tumors. After the erythrocyte lysis, cells were labeled with different antibodies, including CD45-PE (Cat# 103106, Biolegend, San Diego, CA, USA; 1:200), CD11b-APC (Cat# 101212, Biolegend, USA; 1:200), F4/80-APC/Cy7(Cat# 123117, Biolegend, USA; 1:40), CD86-PE/Cy7 (Cat# 105005, Biolegend, USA; 1:40), CD206-BV605 (Cat# 141721, Biolegend, USA; 1:40). The death cells were identified using BD Horizon™ Fixable Viability Stain 510 (FVS510, Cat# 564406, BD Biosciences, Franklin Lakes, NJ,, USA; 1:1000). The data were acquired on a Cytek Aurora/NL spectral flow cytometer (Cytek Biosciences, Fremont, CA, USA) and analyzed using FlowJoTM v10.8.1 software (BD Biosciences, USA). The immunohistochemical staining of tumor tissues was performed following standard protocols to evaluate the expression of ANKRD45 (Cat# Solarbio, Beijing, China; 1:200), AQP3 (Cat# 510926, Zenbio, Chengdu, China; 1:100) and NDUFA4L2 (Cat# 16480-1-AP, Proteintech, Wuhan, China; 1:200). The data were collected by Aperio VERSA 8 (Leica, Wetzlar, Germany) and the positive areas were analyzed by Aperio ImageScope 12.4.6 software (Leica, Germany).

### 3.3. Data Source

The RNA sequencing data and relevant clinical information were downloaded from The Cancer Genome Atlas (TCGA) database. GSE84437/GSE62254 and GSE102556/GSE80655 were obtained from the Gene Expression Omnibus (GEO) database for the validation of the signature and the MDD score.

### 3.4. Transcriptome Sequencing (mRNA-Seq) and Analysis

Total RNA from PDX after 3 weeks of CUMS on the nude mouse was extracted and then analyzed by Singleron Biotechnologies (Nanjing, China). Libraries were constructed according to the NEB normal method. The library was amplified and the total DNA was screened. Then, high-throughput sequencing was performed. Quantitative mapping was performed using HISAT2 and the featureCount algorithm, and DESeq software (version 1.44.0) was used to screen out the different genes according to the thresholds of the adjusted *p*-value < 0.1. Raw data and processed data were uploaded to the GEO database (GSE262056). Next, Kyoto Encyclopedia of Genes and Genomes (KEGG) pathway and gene ontology (GO) enrichment analyses were performed for the differentially expressed genes (DEGs) using the Database for Annotation, Visualization and Integrated Discovery (DAVID) database. Finally, the “MCODE” plugin in Cytoscape was used to analyze the hub module of protein–protein interaction (PPI) obtained from the STRING database.

### 3.5. Analysis of the Gene Expression Patterns in Stomach Adenocarcinoma Dataset

Differentially expressed genes were obtained by comparing 34 normal and 373 stomach adenocarcinoma (STAD) tissues in TCGA, which were considered tumor-related genes, and the filter was |log2 (Foldchange)| > 1 and an adjusted *p* value < 0.05.

### 3.6. Cluster Analysis

We performed cluster analysis using R package “ConsensusClusterPlus” to identify the depression-related molecule subtypes. The prognosis between the two clusters was compared using Kaplan–Meier (KM) analysis. The heatmap was used to show the different gene expression patterns between the two clusters.

### 3.7. MDD Score

The most important gene co-expression module in males with MDD across brain regions, which is called ‘Peru’, was obtained from a previous study. We applied single sample GSEA (ssGSEA) algorithm to calculate the scores of the Peru of each tumor sample as MDD score. The MDD score was significantly higher in the depression group than in the control group, which was validated in GSE102556/GSE80655. K–M analysis was performed to compare the prognosis between high and low MDD score in TCGA–STAD data.

### 3.8. Construction and Validation of the Prognostic Signature

The prognostic signature was constructed using independent genes identified through multivariate Cox regression analysis in STAD, based on 56 hub genes, to predict overall survival after surgery. The risk score was calculated using the following formula, where Coefi represents the coefficients, and xi represents the expression level of the gene (NDUFA4L2, ANKRD45, and AQP3):

$$Risk\;score=\sum_{i=1}^{3}Coefi*xi$$

The best cutoff risk-score obtained by X-tile of the patients with STAD was then used to categorize them into high- and low-risk groups. K-M analysis and a Receiver Operation Characteristic (ROC) curve were used to evaluate the signature’s prognostic value. Then, the signature was validated in GSE84437 and GSE62254. A multivariate Cox analysis of clinical parameters and the signature was performed to identify the independent risk factors. A nomogram was then constructed from the Cox result and judged by calibration plots.

### 3.9. The Hub Genes’ Functional Enrichment Analyses

The hub genes’ co-expression genes in stomach adenocarcinoma were obtained from GEPIA (http://gepia.cancer-pku.cn/, accessed on 12 July 2024). GO enrichment analyses were performed for the top 100 co-expression genes using the DAVID database.

### 3.10. Gene Set Enrichment Analysis (GSEA) Based on the Signature

The software GSEA-4.3 was used to conduct GSEA in different risk groups in the TCGA cohort. Filter conditions were |normalized enrichment score (NES)| > 1.6, nominal *p*-value < 0.05, and FDR q-value < 0.25.

### 3.11. Tumor-Related Scores

Angiogenic activity, mesenchymal–epithelial–mesenchymal-transition (EMT), and tumorigenic cytokines were closely related to tumor development, and their ssGSEA scores are associated with prognosis for gliomas, which was found in previous studies. The relevant marker genes are listed in Table S2. We calculated the above scores using the ssGSEA algorithm for each tumor sample in the TCGA cohort as well.

### 3.12. Immune Landscape Analysis

The ESTIMATE algorithm was used to calculate the stromal score, estimate score, immune score, and tumor purity score in the TCGA cohort. The CIBERSORT algorithm was used to predict the composition of infiltrating immune cells in the TCGA cohort. The prognosis of patients grouped by the composition of infiltrating immune cells was compared using K–M analysis. The tumor immune dysfunction and exclusion (TIDE) score of the TCGA cohort was calculated through the TIDE database.

### 3.13. Chemotherapy Response and Small-Molecule Drugs

Through the Genomics of Drug Sensitivity in Cancer (GDSC) database, we predicted the response of STAD patients to chemotherapy drugs. The R package “pRRophetic” was employed to calculate the half maximal inhibitory concentration (IC50), which served as a metric for evaluating patient response. Additionally, the connectivity map (cMap) database, an application amalgamating small-molecule drugs, gene expression, and disease information, was leveraged. By comparing the upregulated and downregulated genes between low- and high-risk groups, we identified potential drugs capable of inducing or reversing tumor biological processes. With a *p*-value < 0.05 and an enrichment score ranging from −1 to 0, these potential drugs have the potential to become new target candidates for STAD patients. Furthermore, we obtained structural figures of these candidate drugs from the PubChem database.

### 3.14. Cell Culture

A mouse YTN3 gastric cancer cell line was generously provided by Prof. Sachiyo Nomura of the University of Tokyo [9]. YTN3 cells were cultured in DMEM (Gibco, Grand Island, NY, USA) with 10% fetal bovine serum (FBS) (Gibco, Grand Island, NY, USA) and penicillin–streptomycin (Biosharp, Hefei, China). All cells were maintained in a 5% CO_2_ atmosphere at 37 °C.

### 3.15. Real-Time Quantitative Polymerase Chain Reaction (RT-PCR)

Total RNA was extracted using TRIzol reagent (Agbio, Cat# AG21101, Changsha, China), and 1 mg was utilized for reverse transcription with the Hifair II 1st strand cDNA synthesis supermix (Cat# 11120ES60, Yeasen, Shanghai, China). Target gene expression levels were calculated using the delta Ct method and normalized to the housekeeping gene GAPDH. Oligonucleotide primer sequences are provided as follows:

GAPDH-F: CATCACTGCCACCCAGAAGACTG;

GAPDH-R: ATGCCAGTGAGCTTCCCGTTCAG;

NDUFA4L2-F: TGGCTTCATCTGCTTGGGCATG;

NDUFA4L2-R: GTCATTGGGACTCAGGCGGTTC;

ANKRD45-F: ATGGGGCAGAATAGACCCG;

ANKRD45-R: TCTTCATACTCTTGCTGTGAGGA;

AQP3-F: TTTGGCTTCGCTGTCACCCTTG;

AQP3-R: CCAGTGCATAGATGGGCAGCTT;

### 3.16. Statistical Analysis

All statistical analyses and visualizations were conducted using R version 4.3.1 (R Foundation for Statistical Computing, Vienna, Austria). Data comparisons between two groups utilized the Wilcoxon test. The X-tile software (version 3.6, New Haven, CT, USA) was utilized to determine the optimal cut-off for survival analyses. Statistical differences between Kaplan–Meier survival curves were assessed using the log-rank test. Patients with missing information in the patient cohort were excluded. Statistical significance was defined as *p* < 0.05.

## 4. Discussion and Conclusions

In this study, we constructed a gene signature based on the NDUFA4L2, ANKRD45, and AQP3 genes. The gene signature predicted the prognosis of OS, tumor immune dysfunction and exclusion, the infiltration of M2 macrophages and neutrophils, angiogenetic activities, the enrichment of epithelial–mesenchymal transition signaling pathways, depression risk, and activated neurogenesis markers of gastric adenocarcinoma in both the development and validation cohort.

The gene signature was made up of three genes, NDUFA4L2, ANKRD45, and AQP3 genes. NDUFA4L2 is a subunit of the mitochondrial complex I, which serves as a part of the electron transport chain [10]. The dysregulation of NDUFA4L2 adversely affects tissues that require a high level of energy, including the brain and tumors. Induced by hypoxia, NDUFA4L2 may be involved in the metabolic remodeling to promote the development of gastric cancer [11,12]. As a critical metabolic gene, NDUFA4L2 was also a key factor in regulating the immune cells including macrophages by the activation of pro-inflammatory functions [13]. On the other hand, the NDUFA4L2 was reported to be associated with neurodegenerative disease and depression [14,15]. NDUFA4L2 partially contributed to the progress of tumor and depression risk of gastric adenocarcinoma due to the capacity to induce the immune suppressive cytokines and pro-inflammatory factors. Indeed, AQP3 was another stress-response-related gene. As an aquaporin water channel family member, AQP3 is the transporter of small molecules, including water, glycerol, and H_2_O_2_ [16]. AQPs are engaged in other cellular processes like neurodegenerative disease and inflammation [17,18]. Studies previously reported that AQP3-mediated H_2_O_2_ transport and increased intracellular H_2_O_2_ concentration increased inflammation, cell proliferation, and cell migration by acting as a secondary messenger for cell signaling involving factors such as NF-κB and PTEN, acting as an oncogene [17]. In muscle-invasive bladder cancer, the downregulation of AQP3 was reported to be associated with worse progression-free and cancer-specific survival, acting as a tumor suppressor gene [19]. In our study, AQP3 loss was reported to be associated with worse progression-free and cancer-specific survival, acting as a tumor suppressor gene. The AQP3 loss was positively correlated with the worse OS of gastric adenocarcinoma patients. The function of AQP3 needs to be verified with proper models in our future study. In terms of ANKRD45, even though few studies focus on this gene, the limited studies showed that ANKRD45 was involved in cell proliferation, and upregulated in Alzheimer’s disease [20,21]. Based on the functions of these genes in cancer and neurodegenerative disease, it is rational that the gene signature based on NDUFA4L2, ANKRD45, and AQP3 could predict the prognosis and depression risk for gastric adenocarcinoma.

The gene signature divided the gastric adenocarcinoma into different phenotypes. The patients with high scores share the characteristics of high levels of tumor immune dysfunction and exclusion, a higher infiltration of M2 macrophages and neutrophils, higher angiogenetic activities, and more enriched epithelial–mesenchymal transition signaling pathways. Recently, studies have recovered that monocytic myeloid-derived suppressor cells (M-MDSC) and polymorphonuclear MDSC (PMN-MDSC) provide pro-tumorigenic immunosuppressive capacities and resistance to immunotherapy in gastric cancer [22,23,24]. The M2 macrophage and neutrophils are the representative population of M-MDSCs and PMN-MDSCs [25]. In our study, gastric adenocarcinoma patients with high levels of infiltration of M2 macrophages and neutrophils experienced dramatically shorter overall survival. Thus, targeting the M-MDSCs and PMN-MDSCs may be a strategy for combination therapy for gastric cancer. For the molecular characteristics, there are higher angiogenetic activities for gastric adenocarcinoma with a high risk-score. This can be explained by the higher expression of KDR in this subgroup. The hypoxia microenvironment easily stimulates the expression the angiogenesis-related gene expression, including KDR [26,27]. In both the preclinical and clinical studies, anti-angiogenesis can benefit patients with overall survival for gastric cancer [28,29]. The gene signature may identify an advantageous patient population for anti-angiogenesis therapy. Even if it is not surprising, the enriched EMT signaling pathways make the subgroup a more aggressive phenotype. The EMT pathway is a classical approach to trigger distant organ metastasis [30]. In gastric cancer, ETM-related genes like E-cad, N-cad, Vimentin, Snail, Slug, and Twist were deregulated [31]. The changes in these genes are partially driven by the activation of the oncogene or inactivation of the tumor suppressor gene and partially triggered by the interaction between the tumor cells and immune cells. It is hard to directly target the EMT to prevent the metastasis of cancer, and interventions for upstream or immune cells may be a choice.

Based on our previous work about the interaction between gastric cancer and depression, we tried to find potential molecular markers to predict the depressive status of gastric cancer patients. Fortunately, our signature showed promising prediction value for the patients with major depressive disorder. In order to get the potential mechanism, we analyzed the levels of depression-relevant cytokines and confirmed that CCL2, IL-6, and IL-10 significantly increased in the gastric adenocarcinoma with high risk score. These cytokines are transported to the central nervous system (CNS) and activate the resident macrophages to promote the development of MDD [32,33]. Another factor contributing to the depression was the increased levels of macrophages and neutrophils in the subgroup with a high risk-score. Response to the stress, macrophages and neutrophils mobilized from bone marrow to peripheral blood, and the activation of the macrophages and neutrophils released depression-related cytokines [34]. In addition to the cytokines and immune cells, we analyzed the neurogenesis-related genes in the tumor tissue of the subgroups and confirmed that the subgroup with a high risk-score had an active expression of these genes, including GFAP, NES, DPYSL3, S100B, and NCAM1. Even neurogenesis-related genes were suppressed in the CNS in the patients with MDD [35]; the increased levels of neurogenesis related genes might contribute to the tumor-induced neurogenesis in the tumor. As reported previously, tumor-derived neurotrophins and extracellular vesicles attracted adjacent peripheral fibers (autonomic/sensorial) and neural progenitor cells, and the parasympathetic fibers promoted the invasion and metastasis of the tumor [36]. Strikingly, tumor-induced axonogenesis builds more connections between cancer cells and CNS, and the neuropathic pain derived from axonogenesis may partially contribute to the MDD for gastric adenocarcinoma patients [37,38]. The driving force for changes in the neurogenesis genes remains unknown. Even NDUFA4L2, ANKRD45, and AQP3 are involved in the neurodegenerative disease, their functions and relationship with the neurogenesis-related genes need to be assessed.

With these characters identified by the signature, we tried to find out the precision treatment strategy for the subgroups. With the Genomics of Drug Sensitivity in Cancer database and the connectivity map dataset, we predicted that the gastric adenocarcinoma patients at a high-risk level will be sensitive to Pazopanib, XMD8.85, Midostaurin, HG.6.64.1, Elesclomol, Linifanib, AP.24534, Roscovitine, Cytarabine, and Axitinib. Some of these drugs, like Pazopanib, Midostaurin, Axitinib, and Docetaxel, had been employed for the clinical trial and were confirmed to prolong overall survival for several cancer types [39,40,41,42]. More and more researchers are trying to use the psychotropic drugs for the treatment of cancer, and have found that Chlorpromazine, Fluoxetine, and Valproic acid have anti-cancer activities [43]. Importantly, Yang’s team confirmed that antidepressive drug monoamine oxidase A inhibitor phenelzine treatment significantly suppressed tumor growth in preclinical mouse syngeneic and human xenograft tumor models in a T cell-dependent manner. These findings suggest that combination therapy with antidepressive drugs and anticancer drug may have potential higher efficiency in cancer therapy [44]. Our prediction system took all the factors identified by the gene signature into account; we believe it provides us with indications about the treatment strategy for gastric adenocarcinoma, and offers us directions for the basic and clinical research for gastric adenocarcinoma with MDD. While we must acknowledge that our limitation lies in the lack of support from preclinical and clinical data, we hope we can achieve a breakthrough based on our findings in this study, and provide more details for cancer patients with depressive disorder.

## Data Availability

Available at https://tau.amegroups.com/article/view/10.21037/tau-23-483/dss.

## Supplementary Materials

The following supporting information can be downloaded at: https://www.mdpi.com/article/10.3390/ijms251910776/s1.
