# Supplementary figures and images for: Development and Validation of a Comprehensive Prognostic and Depression Risk Index for Gastric Adenocarcinoma
Source: Int J Mol Sci. 2024 Oct 7;25(19):10776. doi: 10.3390/ijms251910776 (PMC11476876; doi:10.3390/ijms251910776)

## Slide 1
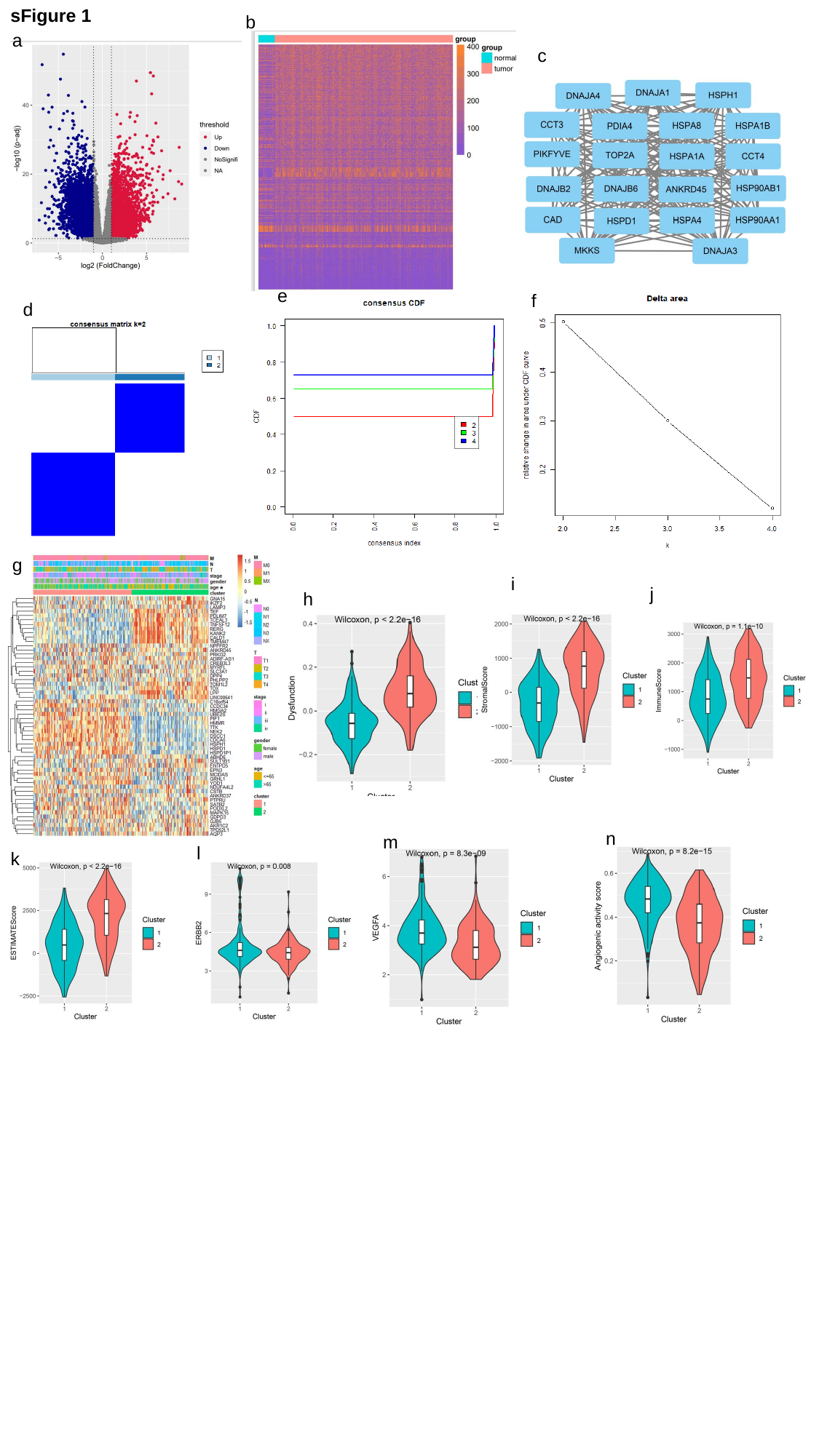

sFigure 1
b
a
c
e
f
d
g
i
j
h
n
m
l
k

## Slide 2
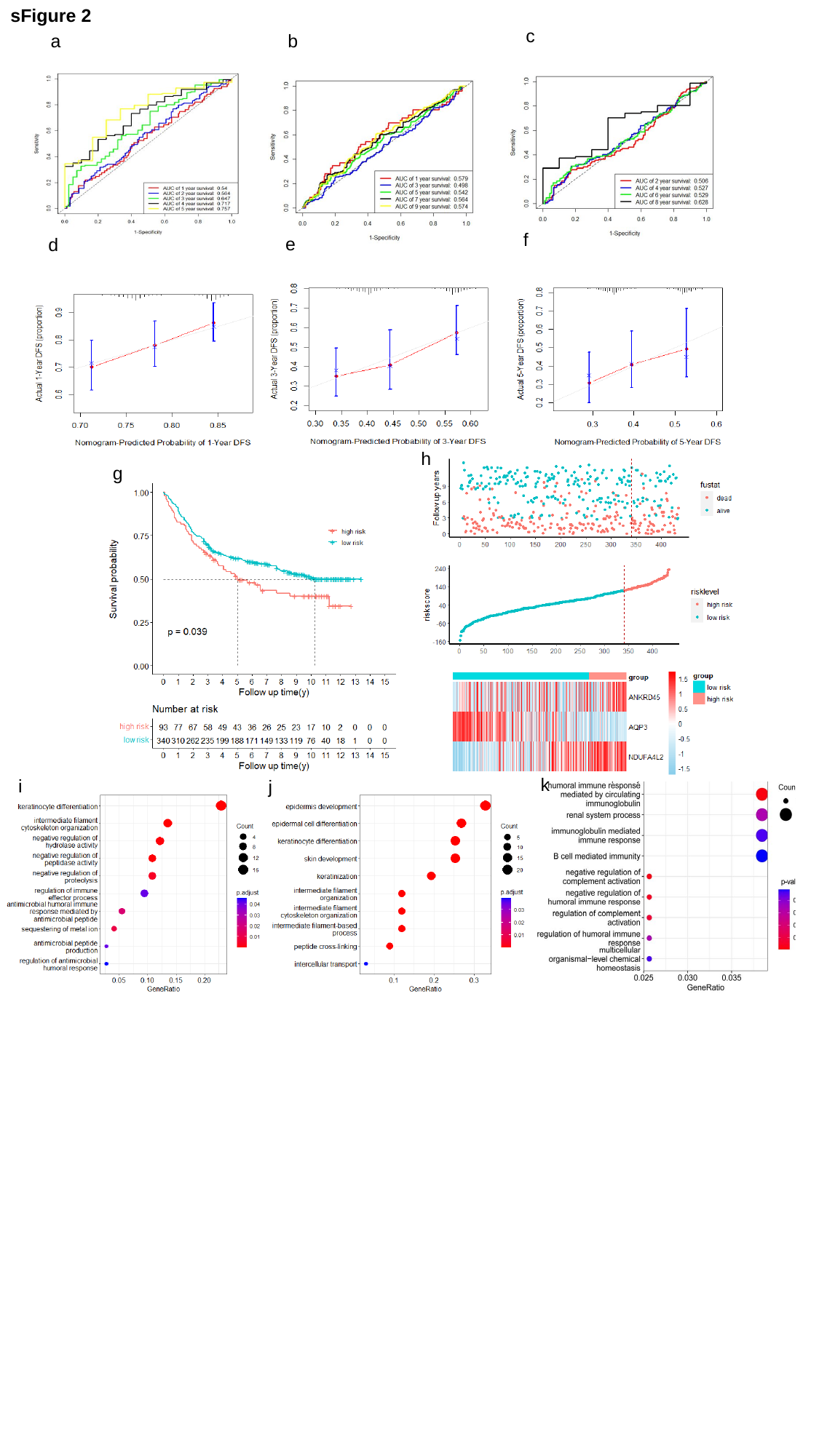

sFigure 2
c
b
a
f
e
d
h
g
k
i
j

Supplement: Supplementary file 1 [file ijms-25-10776-s001.zip › Supplementary figures.pptx]
